# Asymptomatic azygos vein overflow in a young patient with primary mediastinal seminoma

**DOI:** 10.1111/1759-7714.13170

**Published:** 2019-09-30

**Authors:** Raimondo Di Liello, Francesca Sparano, Maria Lucia Iacovino, Giuseppe Viscardi, Carminia Maria Della Corte, Andrea Ronchi, Rosa Laura Sabetta, Morena Fasano, Giovanni Vicidomini, Alfonso Reginelli, Fortunato Ciardiello, Floriana Morgillo

**Affiliations:** ^1^ Medical Oncology, Precision Medicine Department Università degli Studi della Campania Luigi Vanvitelli Naples Italy; ^2^ Pathology Unit, Department of Mental and Physical Health and Preventive Medicine Università degli Studi della Campania Luigi Vanvitelli Naples Italy; ^3^ Thoracic Surgery Unit Università degli Studi della Campania Luigi Vanvitelli Naples Italy; ^4^ Department of Internal and Experimental Medicine Institute of Radiology, Università degli Studi della Campania Luigi Vanvitelli Naples Italy

**Keywords:** Primary mediastinal seminoma, radiology, superior vena cava syndrome

## Abstract

The azygos system is the most important pathway for decompression of the superior vena cava (SVC) when a blood flow obstruction to the right atrium is present. Thoracic and mediastinal malignancies, mainly lung cancers, are responsible for 60%–85% of superior vena cava syndrome (SVCS) cases. An uncommon origin of SVCS is primary malignant mediastinal germ cell tumor (PMMGCT) which represent 1%–4% of all mediastinal tumors and can be divided into two broad groups: seminomas and nonseminomatous germ cell tumors (NSGCTs). Primary mediastinal seminomas clinical presentation is often nonspecific, even if the majority of patients present with superior vena cava involvement. Here, we present the radiologic features of asymptomatic azygos system overflow in a patient with primary mediastinal seminoma.

## Introduction

Superior vena cava syndrome (SVCS) occurs when a blood flow obstruction through the superior vena cava (SVC) to the right atrium exceeds the compensatory ability of collateral blood vessels.^1^ The azygos system is the most important pathway for decompression of an SVC obstruction.^2^ Thoracic and mediastinal malignancies, mainly lung cancers, are responsible for 60%–85% of SVCS cases.^3^ Here, we present a case of azygos vein overflow in a 19‐year‐old patient with asymptomatic primary mediastinal seminoma.

## Case report

**Figure 1:**
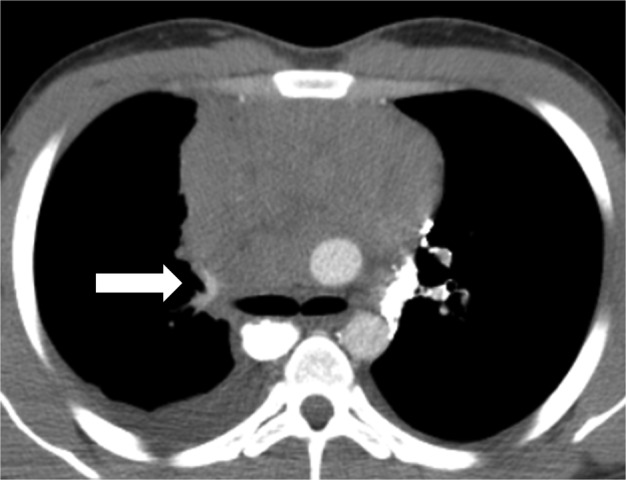
Axial view of baseline computed tomography (CT) scan that showed superior vena cava (SVC) complete collapse due to the huge seminoma mass at the height of carina (arrow).

**Figure 2:**
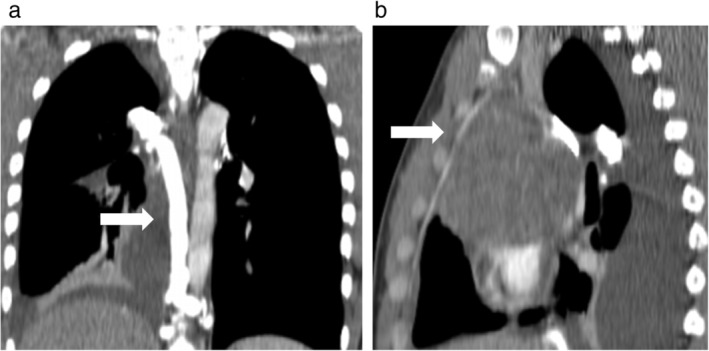
Collateral venous system seen at baseline CT scan. (**a**) Enlarged azygos vein (arrow) reached the diameter of the adjacent thoracic aorta (coronal view, multiplanar reconstruction). (**b**) Internal mammary vein (arrow) wasnot affected by collateral overflow (sagittal view, multiplanar reconstruction).

**Figure 3:**
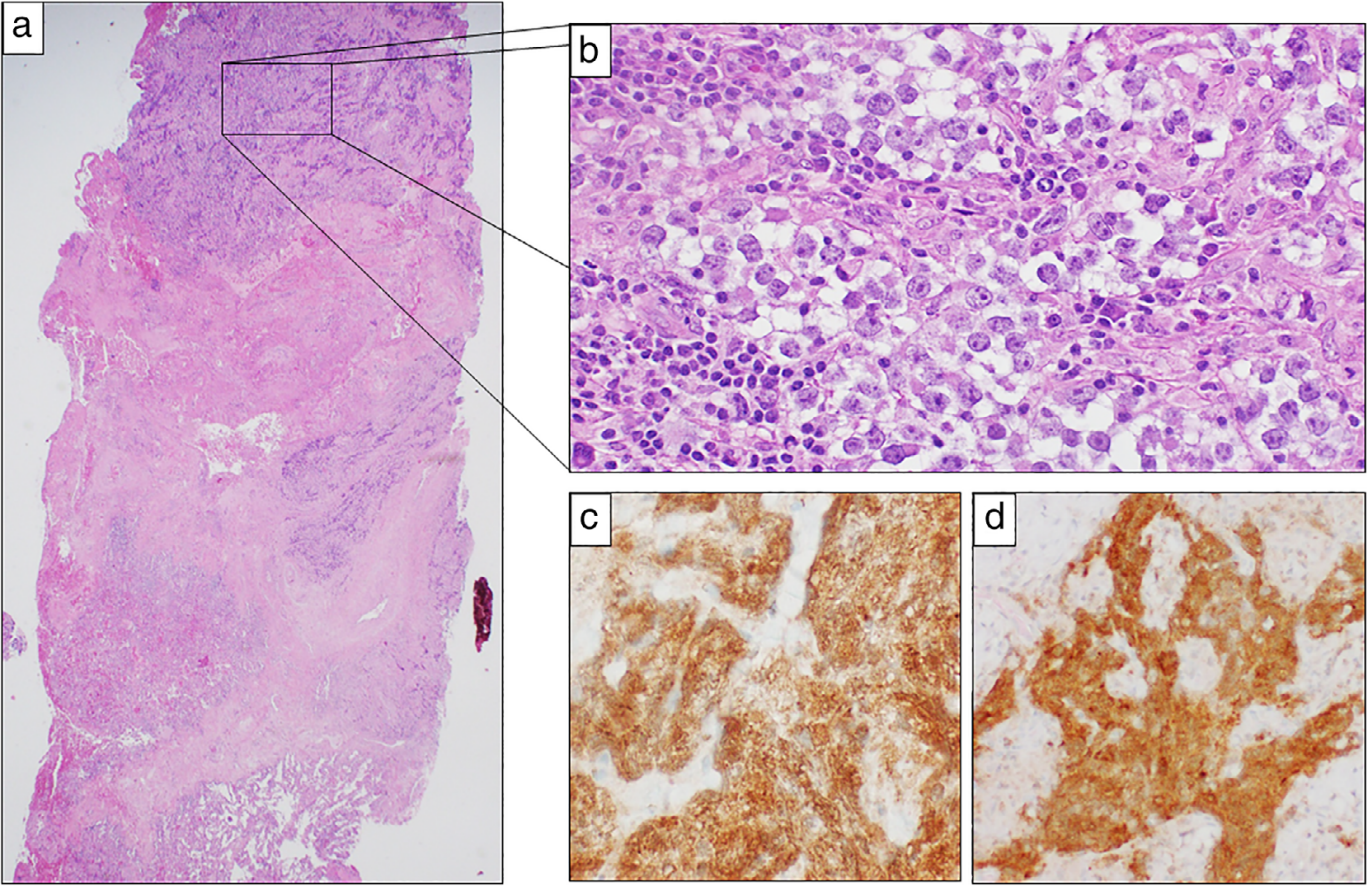
Histological and immunohistochemical features. (**a**) A fibrous fragment with neoplastic cells, embedded in an inflammatory background. The crushing artefact is evident. At the bottom of the section, pulmonary tissue is focally present (hematoxylin and eosin, 2.4×). (**b**) The neoplastic cells show abundant clear cytoplasm and prominent nucleoli (hematoxylin and eosin, 20×). The neoplastic cells expressed PLAP (**c**) and CD117 (**d**) (immunostaining, 20×).

A 19‐year‐old male patient presented to our Institution with evidence of a mediastinal mass after a plain radiograph was performed for a different reason. No symptoms were reported at the colloquium. Noncontrast enhanced computed‐tomography (CT) scan revealed a wide upper mediastinal lesion measuring 11 cm in maximum diameter, adjacent to the aortic arch and compressing the main vessels of the upper mediastinum. o jugular vein enlargement, facial, neck or upper extremity swelling, neurological signs, cough or dyspnea were detected on physical examination. A contrast enhanced CT scan performed prior to biopsy procedure detected a preazygos SVC obstruction.^4^ A mediastinal mass compressed the main bronchus and vessels of upper mediastinum (Fig 1); the superior vena cava (SVC) had totally collapsed at the height of the carina. Collateral venous system overflow enlarged the image of the azygos vein that reached the diameter of thoracic aorta (Fig. 1(a)) without affecting internal mammary vein flow (Fig. 1(b)). Baseline radiological assessment was negative for metastatic disease and considering that complete surgical resection was not viable due to the tumor dimensions and its adherence to the major vessels, transthoracic biopsy was performed. The sampled tissue was formalin fixed, paraffin embedded and hematoxylin/eosin stained. Microscopic examination revealed fibrous tissue fragments, with dense lymphoid and granulomatous inflammatory infiltrate, and with diffuse crushing artefacts. In this context, a neoplastic population was present, organized in lobular aggregates and cellular strands. Neoplastic cells were large‐sized, with abundant clear cytoplasm, roundish nuclei and prominent nucleoli. Immunohistochemistry demonstrated positivity for placental alkaline phosphatase (PLAP) and CD117, and negativity for cytokeratin AE1/AE3, p63 and CD20. A final diagnosis of seminoma was rendered (Fig. 2). At baseline, serum α‐fetoprotein (AFP) was normal, lactate dehydrogenase (LDH) was only slightly increased and β human chorionic gonadotoropin (HCG) reached 2000 mIU/mL. A diagnosis of primary mediastinal seminoma was made. In accordance with international guidelines, the patient underwent four cycles of BEP protocol five‐days chemotherapy (cisplatin 20 mg/m^2^ day 1 to 5, etoposide 100 mg/m^2^ day 1 to 5, bleomycin 30 IU/days 2, 9, 16). After the first cycle of therapy, serum β‐HCG reduced to 20 IU/L (local LLN <10 mIU/mL) and after the fourth cycle was negative (<0.3 mIU/mL). Moreover, CT scan radiological evaluation performed after four cycles of chemotherapy showed a partial response (PR) per RECIST 1.1 with a significative reduction in the mediastinal mass (Fig. 3).Unfortunately, due to the adherence of the tumor to the right atrium, complete surgical resection was considered not feasible.

**Figure 4:**
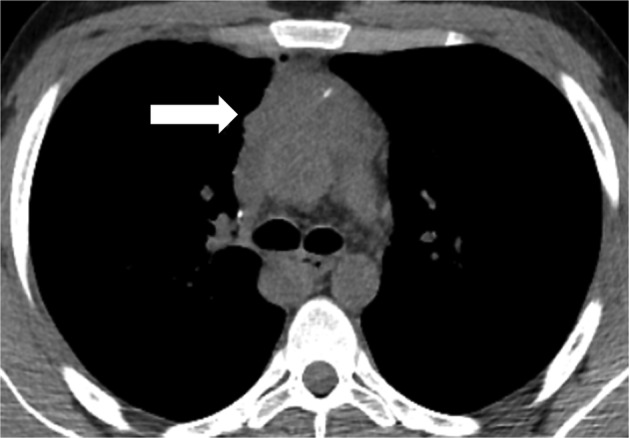
Axial view of CT scan evaluation performed after four cycles of chemotherapy according to BEP protocol. Partial response (PR) per RECIST 1.1 was reached with a significant decrease of tumor mass (arrow) and a reduction in main bronchus compression.

## Discussion

Primary malignant mediastinal germ cell tumors (PMMGCTs) represent 1%–4% of all mediastinal tumors,^5^ PMMGCTs usually arise in the mediastinum followed by pineal gland, retroperitoneum and sacrococcygeal area and can be divided into two broad groups: seminomas and nonseminomatous germ cell tumors (NSGCTs), this category includes teratocarcinomas, yolk sac tumor, embryonal carcinoma, choriocarcinoma, and mixed tumors.^6^, ^7^ Primary mediastinal seminomas occur mostly in men of the second to fourth decades and initial clinical presentation is often nonspecific and even if a major part of patients present with SVC involvement, only 10% of them experience signs and symptoms of SVCS.^8^ In our case report, we presented radiologic feature of azygos system overflow in a patient with primary mediastinal seminoma that at baseline assessment did not present any signs or symptoms of SVCS. Notably, clinical presentation of superior vein cava blockage depends on the speed, severity and location of the obstruction. With acute obstruction, symptoms may be severe, including swelling of the subcutaneous tissues of head and neck, facial flushing, bilateral upper extremity swelling, neurological signs, dyspnea, headache, and cough. On the contrary, obstructions that arise slowly may help to develop collateral drainage with no or only mild symptoms.^9^ Moreover, in our case, CT scan showed only azygos system involvement with normal internal mammary veins. This may be related to the level of the obstruction: when the vein flow is blocked at a lower level and the azygos vein is also obstructed, the collateral circulation establishes a communication between the SVC and inferior vena cava (IVC) via minor communicating channels as internal mammary veins or superior and inferior epigastric veins to iliac veins and then to IVC.^10^ In conclusion, the clinical and radiologic features in our patient may reflect these two aspects of SVC blockage; on the one hand the speed of obstruction development and on the other hand, its level. The young age of the patient could explain the rapid adaptation of the thoracic venous system that resulted in azygos vein overflow and CT scan image enlargement without clear clinical evidence of SVCS.
